# Sustained delivery of calcium and orthophosphate ions from amorphous calcium phosphate and poly(L-lactic acid)-based electrospinning nanofibrous scaffold

**DOI:** 10.1038/srep45655

**Published:** 2017-03-31

**Authors:** Xufeng Niu, Zhongning Liu, Feng Tian, Siqian Chen, Lei Lei, Ting Jiang, Qingling Feng, Yubo Fan

**Affiliations:** 1 Key Laboratory for Biomechanics and Mechanobiology of Ministry of Education, School of Biological Science and Medical Engineering, Beihang University, Beijing 100191, China; 2 Department of Prosthodontics, Peking University School and Hospital of Stomatology; National Engineering Laboratory for Digital and Material Technology of Stomatology; Beijing Key Laboratory of Digital Stomatology, Beijing 100081, China; 3 State Key Laboratory of New Ceramic and Fine Processing, Tsinghua University, Beijing 100084, China; 4 National Research Center for Rehabilitation Technical Aids, Beijing 100176, China

## Abstract

The purpose of this study is to investigate electrospinning poly(L-lactic acid) (PLLA) nanofibrous scaffold with different contents of amorphous calcium phosphate (ACP), which is suitable for using in bone regeneration through sustained release of calcium and orthophosphate ions. Three groups of nanofibrous scaffolds, ACP-free PLLA, ACP-5 wt%/PLLA and ACP-10 wt%/PLLA, are developed and characterized by scanning electron microscopy and gel permeation chromatography. Calcium and phosphate colorimetric assay kits are used to test ions released from scaffold during hydrolytic degradation. The results show ACP-5 wt%/PLLA and ACP-10 wt%/PLLA scaffolds have relatively high degradation rates than ACP-free PLLA group. The bioactivity evaluation further reveals that ACP-5 wt%/PLLA scaffold presents more biocompatible feature with pre-osteoblast cells and significant osteogenesis ability of calvarial bone defect. Due to the facile preparation method, sustained calcium and orthophosphate release behavior, and excellent osteogenesis capacity, the presented ACP/PLLA nanofibrous scaffold has potential applications in bone tissue engineering.

Hydroxyapatite (HA) is an essential mineral component in natural bone tissue, which has been extensively used as osteoconductive material in bone regeneration^1^,^2^,^3^,^4^. It provides mechanical strength to bone and serves as storage for mineral ions (mainly calcium and phosphate groups). These ions play critical role in biomineralization of bone matrix^5^,^6^,^7^,^8^. On the other hand, the highly crystalline HA is usually insoluble and commonly lacks osteoinductive potential, which hinders new bone ingrowth and integration with the native bone.

During the biomineralization of natural bone tissue, the initial formed solid phase is amorphous calcium phosphate (ACP), which further converts into HA within organic matrix^9^,^10^. ACP is thermodynamically unstable and tends to spontaneously transform to crystalline calcium phosphate^11^. Such instability and easy transformation towards crystalline phase are of great biological relevance. Specifically, the initiating role ACP plays in matrix vesicle biomineralization raises its importance as a pivotal intermediate in skeletal calcification^12^. Due to its significant chemical and structural similarities with calcified tissues, as well as fine biocompatibility and bioresorbability, ACP is a very promising candidate for manufacture of artificial bone grafts^13^,^14^,^15^,^16^.

During the conversion of ACP to crystalline phase, it always accompanied with calcium and orthophosphate ions release^17^,^18^,^19^. According to the previous study, these ions could facilitate osteointegration of artificial bone graft through the formation of a thin calcium phosphate layer at the graft-host interface^20^,^21^,^22^. For example, some *in vitro* studies suggested that supplementing cell culture medium with calcium and phosphate ions could stimulate osteoblast differentiation and biomineralization^23^,^24^,^25^,^26^,^27^. *In vivo* studies also demonstrated that phosphate containing hydrogels had ability to facilitate bone growth within a critical-size cranial defect^28^,^29^. Based on these backgrounds, it is rational to deduce that ACP has possibility to achieve the enhanced bone regeneration and osteointegration over the insoluble crystalline calcium phosphates since it can serve as a vehicle to deliver calcium and orthophosphate ions.

However, to date, ACP is rarely used as a graft for bone regeneration mainly because of its rapid dissolution and release of inorganic ion, leading to the diminished mechanical properties^17^. Hybridizing ACP with polymer matrix is a promising choice to overcome these defects^30^,^31^. Here, we develop ACP particle and poly(L-lactic acid) (PLLA) based nanofibrous scaffold by electrospinning method, aiming to achieve the long-term and adjustable delivery of calcium and orthophosphate ions. The composite scaffold is characterized by scanning electron microscopy (SEM). Its degradation and ACP transformation are examined by gel permeation chromatography (GPC) and colorimetric assay accordingly.

## Results and Discussion

### Characterization of electrospinning nanofibrous scaffold

To get ACP and PLLA-based electrospinning nanofibrous scaffold, firstly ACP was prepared using a wet chemical method. X-ray diffraction (XRD) pattern revealed no discernable peaks of crystalline calcium phosphate but a characteristic hump of amorphous phase at around 30° (Fig. S1, Supplementary Information). Transmission electron microscopy (TEM) micrograph further showed that the sample was consisted of nanoparticles with diameters in the range of 50 to 100 nm. Selected area electron diffraction indicated a typical diffraction pattern of amorphous halo ring, which was consistent with the XRD result.

**Figure 1:**
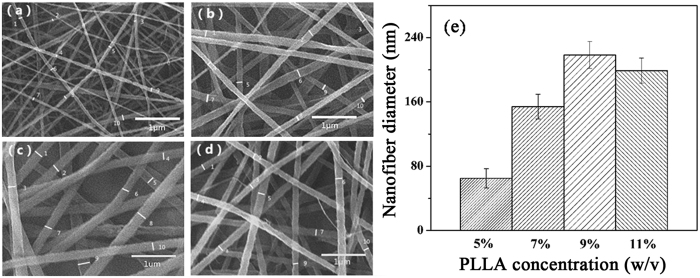
SEM micrographs of electrospinning ACP-free PLLA nanofibrous scaffold with different PLLA concentrations. (**a**) 5%, (**b**) 7%, (**c**) 9%, (**d**) 11%. The corresponding average diameter of nanofibers was evaluated using Image J software (n = 10) (**e**).

Thinking that polymer concentration is the main factor to determine fibrous diameter, various PLLA concentrations were investigated. Figure 1 shows the diameter distribution of ACP-free PLLA electrospinning scaffolds with changing polymer concentrations. Increasing PLLA concentration from 5% to 9%, the nanofiber diameter increased nearly 2 times accordingly. Further improving PLLA concentration to 11% was not accompanied with the increasing nanofiber diameter. As a result, PLLA concentration of 9% was chose in the following study, which could provide suitable nanofiber facilitative for apatite mineral encapsulation.

**Figure 2:**
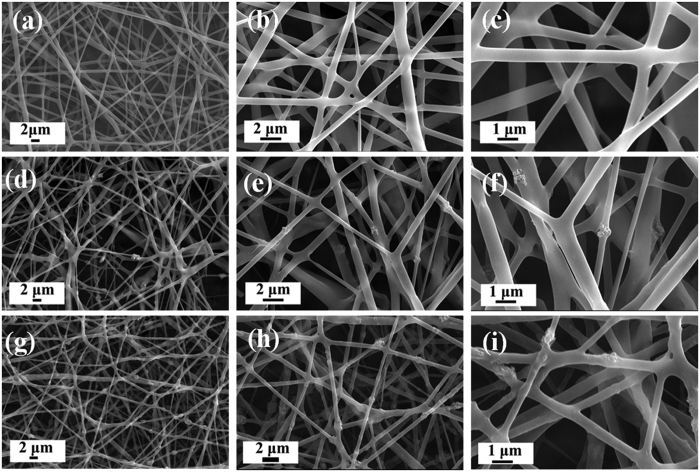
SEM micrographs of PLLA-based electrospinning nanofibrous scaffold with different contents of ACP particles. (**a–c**) Morphology of ACP-free PLLA group. (**d–f**) Morphology of ACP-5 wt%/PLLA group. (**g–i**) Morphology of ACP-10 wt%/PLLA group.

Figure 2 is the morphology of PLLA-based nanofibrous scaffold with 3 different contents of ACP particles. Compared with smooth surface of ACP-free PLLA scaffold, both ACP-5 wt%/PLLA and ACP-10 wt%/PLLA showed rough surface morphology. Besides, increasing ACP content from 5% to 10% accompanied with more uneven surface and some of ACP particles further gathered together to form aggregation.

### *In vitro* hydrolytic degradation

#### Weight and molecular weight change

**Figure 3:**
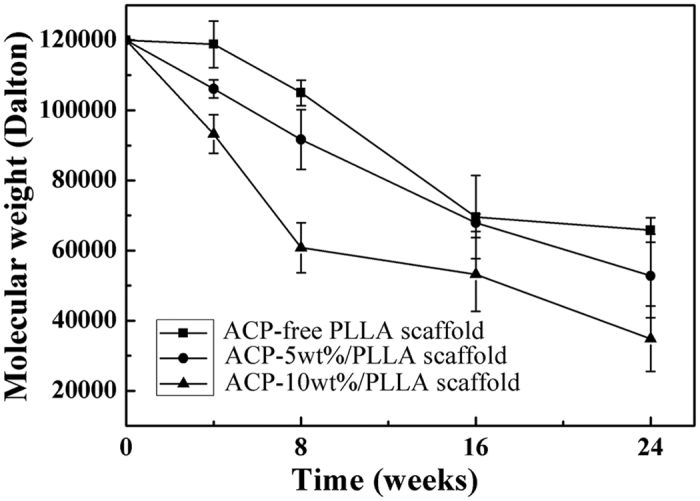
Time dependence of number average molecular weight decrease for ACP/PLLA electrospinning nanofibrous scaffolds with different contents of ACP particles during 24 weeks of *in vitro* hydrolytic degradation.

To determine the degradation behavior of PLLA-based nanofibrous scaffold, the weight and molecular weight change of PLLA were examined. During 24 weeks of *in vitro* hydrolytic degradation, although the degradation rates of ACP-5 wt%/PLLA and ACP-10 wt%/PLLA scaffolds were higher than that of the control group, all scaffolds still could preserve their initial weight and the degradation rates were less than 10% for all 3 groups (Fig. S2, Supplementary Information). Compared with this slow weight loss, the molecular weight decreased gradually for all 3 kinds of scaffolds (Fig. 3). Moreover, with the presence of ACP in the scaffold and the increase of its content, the molecular weight change became more obvious. As a result, after degrading for 24 weeks, the molecular weight decreases for ACP-5wt%/PLLA and ACP-10 wt%/PLLA scaffolds were about 56.0% and 71.0% respectively, whereas that for the control group was 45.1%.

The experiment showed that ACP/PLLA nanofibrous scaffolds had relatively high degradation behavior than the control group, especially molecular weight change. According to the early study, PLLA is a strong hydrophobic polymer. Its degradation occurs via a simple hydrolysis of ester backbone and the molecular weight decrease happens prior to the weight loss^32^,^33^. Moreover, ACP is unstable in aqueous solution and tends to crystallize into HA^34^,^35^. Therefore, the hybridization of ACP particles with PLLA matrix could improve the hydrophilicity of composite scaffold and facilitate the degradation under aqueous condition.

#### Ion release

**Figure 4:**
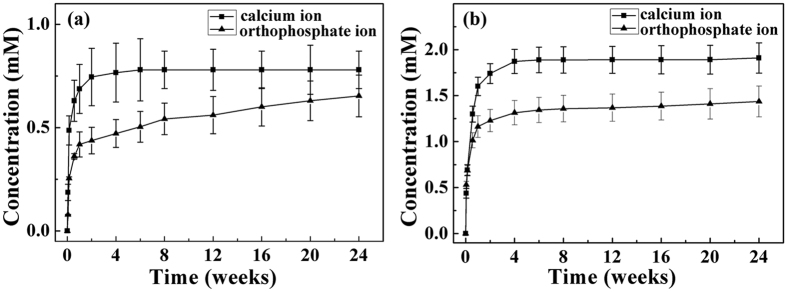
Kinetic release of calcium and orthophosphate ions from ACP/PLLA electrospinning nanofibrous scaffold during 24 weeks of *in vitro* hydrolytic degradation. (**a**) ACP-5 wt%/PLLA scaffold. (**b**) ACP-10 wt%/PLLA scaffold.

Figure 4 is the kinetic release of calcium and orthophosphate ions from ACP/PLLA nanofibrous scaffold. In ACP-5 wt%/PLLA group, both of ions exhibited a biphasic fashion, characterized by a fast release phase at initial 1 week, followed by a slower one at the remaining period of time (Fig. 4a). The concentrations of calcium and orthophosphate ions, released at the initial phase, were 0.69 and 0.42 mM, respectively. Subsequently, the release rates for both of ions gradually decreased as time passed away. Figure 4a also revealed that, the release amount of calcium ion from ACP-5 wt%/PLLA scaffold was higher than that of orthophosphate ion over the whole time period of experiment, which might be related with the transformation of ACP towards HA. As a result, the concentration of calcium ion was 0.80 mM at the end of 24 weeks of *in vitro* hydrolytic degradation, whereas the concentration of orthophosphate ion was 0.65 mM at the same time point. The ACP-10 wt%/PLLA scaffold exhibited the similar release behavior of calcium and orthophosphate ions, except the enhanced concentrations for both of ions, which were consistent with the increasing ACP amount in the scaffold (Fig. 4b). Hence, by changing ACP content in the composite scaffold, the ion release could be easily manipulated.

#### Morphology variation

**Figure 5:**
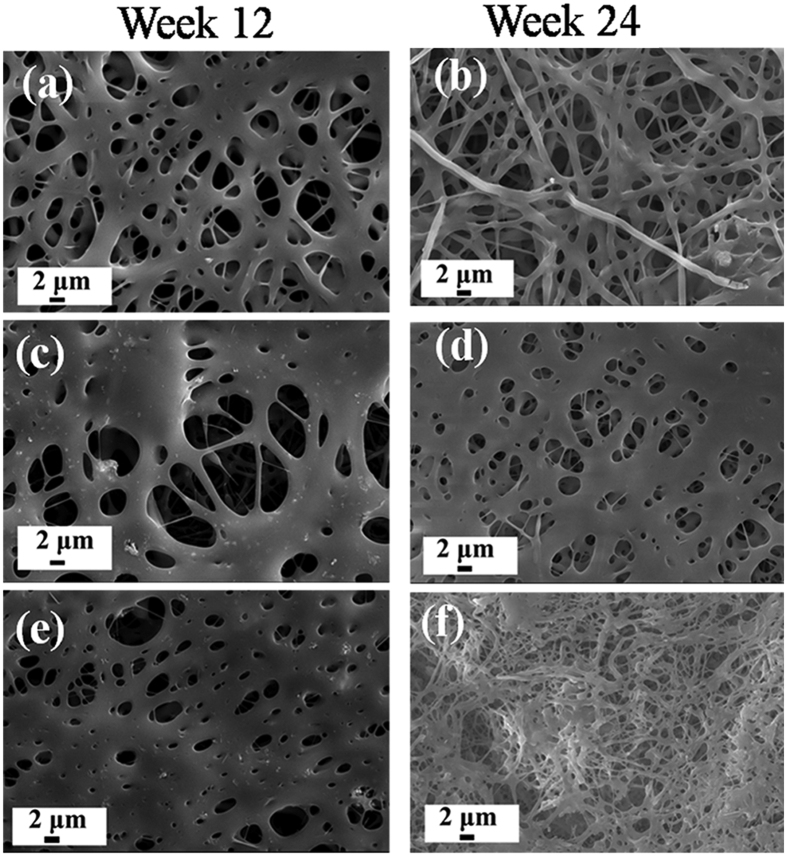
SEM micrographs of ACP/PLLA electrospinning nanofibrous scaffold with various ACP contents during 12 and 24 weeks of *in vitro* hydrolytic degradation. (**a–b**) ACP-free PLLA scaffold; (**c–d**) ACP-5 wt%/PLLA scaffold; (**e–f**) ACP-10 wt%/PLLA scaffold.

Figure 5 shows the morphology evolution of ACP/PLLA nanofibrous scaffold after 12 and 24 weeks of *in vitro* hydrolytic degradation. Overall, compared with the initial nanofibrous morphology as shows in Fig. 2, such structure turned poor in all 3 groups with time went by and this kind of change looked more serious in 2 experimental groups. Moreover, ACP-free PLLA scaffold exhibited the smooth surface during the whole period of hydrolytic degradation, whereas there were many microbumps and microholes appeared on the surface of ACP-5 wt%/PLLA and ACP-10 wt%/PLLA scaffolds, which should attribute to the introduction of ACP. Since ACP is hydrophilic, it could serve as the point to initiate the degradation of PLLA during it transformed into HA in hydrolytic medium. In addition, the fibrous structure appeared partially in ACP-10 wt%/PLLA group after 24 weeks of degradation (Fig. 5f), which should be related with the degradation of external PLLA scaffold. As a result, the inner fibrous structure was exposed and presented the morphology as showed in Fig. 5f.

### *In vitro* biocompatibility evaluation

**Figure 6:**
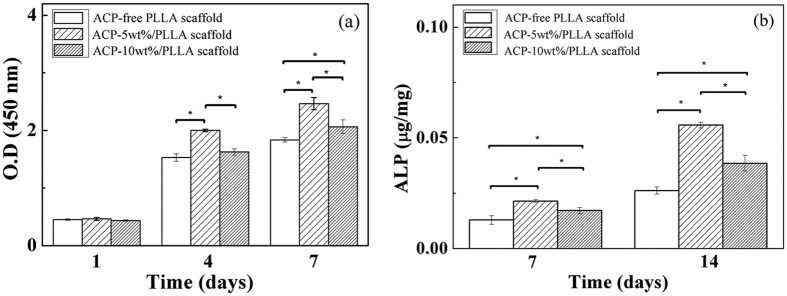
*In vitro* biocompatibility evaluation of ACP/PLLA electrospinning nanofibrous scaffolds with various ACP contents. (**a**) The proliferation of MC3T3-E1 cells cultured with ACP-free PLLA, ACP-5 wt%/PLLA and ACP-10 wt%/PLLA scaffold for 1, 4 and 7 days, respectively. (**b**) ALP activity to show the differentiation of MC3T3-E1 cells cultured with ACP-free PLLA, ACP-5 wt%/PLLA and ACP-10 wt%/PLLA scaffold for 7 and 14 days, respectively. *p < 0.05 was considered to be statistically significant.

The *in vitro* biocompatibility of ACP/PLLA nanofibrous scaffolds was evaluated using pre-osteoblast MC3T3-E1 cells. The morphology observation revealed that a large number of cells appeared on all 3 scaffolds surfaces and most of cells spread well and distributed uniformly throughout the scaffolds after 7 days of incubation (Fig. S3, Supplementary Information). Further proliferation behavior of cells co-cultured with various scaffolds indicated that the cell amounts increased in all 3 groups as incubating time extended from 1 to 7 days (Fig. 6a). The 2 experimental groups presented the better cell proliferation than the ACP-free PLLA scaffold. Moreover, compared with the ACP-10 wt%/PLLA group, the ACP-5 wt%/PLLA scaffold maintained the faster cell growth, especially in the assay of 4 and 7 days (*p* < 0.05). These results illustrated that PLLA nanofibrous scaffolds containing appropriate contents of ACP particle might have the potential to stimulate pre-osteoblast proliferation.

ALP activity, an early marker of osteoblast phenotype, is usually up-regulated at early stage of osteoblastic differentiation^36^. In this study, ALP activity of MC3T3-E1 cells was also assayed in order to determine the cell differentiation ability of 3 nanofibrous scaffolds. As revealed in Fig. 6b, the ACP-5 wt%/PLLA scaffold showed the greatest ALP activity among all 3 groups at both 7 and 14 days of cultivation. The ACP-10 wt%/PLLA scaffold also showed the greater ALP activity than the control group. These results should attribute to the effect of ACP in the scaffold. A suitable ACP amount could promote ALP expression and cell differentiation, whereas such promoting effect turned weak once its amount exceeded the certain content.

### *In vivo* osteogenesis evaluation

**Figure 7:**
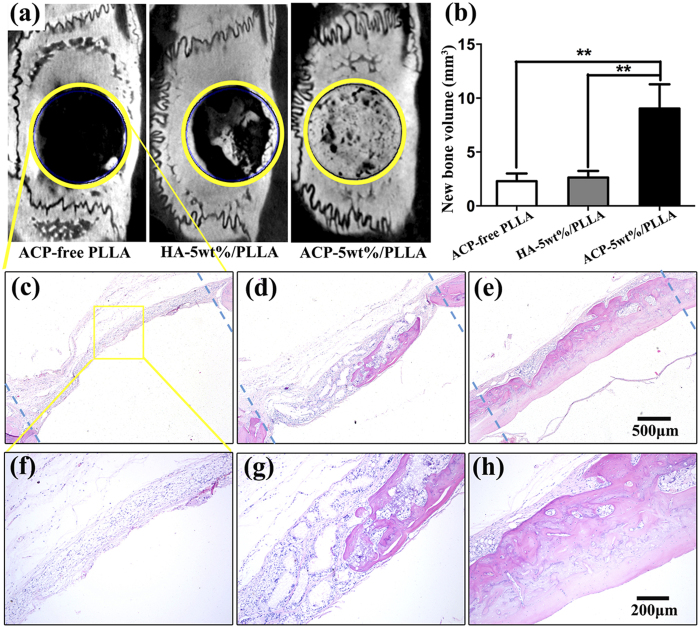
Implantation of cell-free ACP-5 wt%/PLLA scaffold improved bone formation in calvarial defect of rats after 8 weeks. The results were investigated by micro-CT and H&E staining. (**a,b**) Micro-CT images and quantitative micro-CT analysis (new bone volume) showed the regenerated bone volumes. The neo bone volume in ACP-5 wt%/PLLA group was significantly increased than the ACP-free PLLA or HA-5 wt%/PLLA group. (**c–h**) H&E staining showed the calvarial defect and middle area. (**c** and **f**) ACP-free PLLA group almost had no regenerated bone in bone defect area. (**d** and **g**) In HA-5 wt%/PLLA group, a part of bone defect was filled with neo bone. (**e** and **h**) With implantation of ACP-5 wt%/PLLA scaffold, almost total bone repairing was observed in calvarial defect area.

To evaluate the efficiency of electrospinning nanofibrous scaffold to bone defect regeneration, the cell-free ACP-5 wt%/PLLA scaffold was further implanted in 5-mm calvarial bone defect, ACP-free PLLA and HA-5 wt%/PLLA scaffolds implanted rats with the same defect were used as control. After 8 weeks, all specimens were harvested for micro-computed tomography scanning (Micro-CT) and histological analyses by hemotoxylin and eosin (H&E) staining. At 8 weeks after implantation, micro-CT images showed there was almost no regenerated bone in the bone defect area in ACP-free PLLA group and only a small amount of neo bone was formed in HA-5 wt%/PLLA group. In contrast, when ACP-5 wt%/PLLA scaffold was implanted in the bone defects, significant regeneration took place (Fig. 7a). Further information of the new formed bone structure was revealed by histological data. In ACP-5 wt%/PLLA group, Fig. 7e and h showed the new formed calvarial bone was filled with trabecular bone and there was no evidence of any remnants of ACP/PLLA composite. Moreover, the new regenerated bone was integrated with the original bone at the defect margins. However, in HA-5 wt%/PLLA group (Fig. 7d and g), only a small quantity of bone like tissues was visible in the defect area, and the porous like region showed that the scaffold was not totally degraded. These findings suggested that ACP/PLLA scaffold had the higher degradation rate and osteogenic efficiency than the other 2 groups.

The release of calcium and orthophosphate ions from ACP provided a favorable micro-environment for stem cell migration, proliferation and differentiation. Due to the remarkable extracellular matrix mimicking surface structure, ACP/PLLA nanofibrous scaffold created a platform for osteoblasts attachment and matrix mineralization. Additionally, the suitable degradation rate of ACP/PLLA scaffold could improve the osseointegration of the regenerated bone with the host tissue and limit the inflammation or foreign-body reaction. All in all, the cell-free ACP/PLLA scaffold provides an ideal structure and platform for bone repair. Our results clearly suggest that ACP/PLLA electrospinning nanofibrous scaffold has broad potential in clinical application of bone defect regeneration.

## Conclusions

ACP/PLLA nanofibrous scaffold has been developed via a facile electrospinning method. Such scaffold can mimic the architecture of natural bone extracellular matrix and contain water soluble ACP nanoparticles and biodegradable PLLA matrix, which are capable of delivering calcium and orthophosphate ions in a sustained manner and providing a more biocompatible interface for bone defect regeneration. The presented ACP/PLLA nanofibrous scaffolds have potential applications in material induced bone regeneration.

## Methods

### Synthesis of ACP

ACP was synthesized using a wet chemical method^11^. Two kinds of solutions were prepared individually by dissolving 2.95 g of Ca(NO_3_)_2_·4H_2_O and 0.99 g of (NH_4_)_2_HPO_4_ in 20 mL of distilled water and then mixed together. The diluted aqua ammonia was used to adjust pH to 7.4 during this reaction. The reaction product was centrifuged immediately and distilled water was used to wash the precipitate to remove any unreacted ions. The precipitate was collected for freeze-drying process using a SP Scientific VirTis Advantage XL-70 freeze dryer (USA) and characterized by XRD (Rigaku D/Max, Japan) and TEM (JEOL JEM-2100, Japan).

### Preparation of electrospinning nanofibrous scaffold

PLLA was obtained from Evonik Industries AG (Germany) and its nanofibrous scaffold was prepared by electrospinning method. To get an optimized concentration, viscous PLLA solutions with concentrations of 5%, 7%, 9%, 11% (w/v) were prepared by dissolving PLLA in dichloromethane and N-N dimethylformamide mixed solvent at a ratio of 7: 3 (v/v). The solutions were loaded into the syringe and the electrodes were clipped onto the needle. The electrospinning was carried out at a voltage of 20 kV and a flow rate of 0.4 mL/h. The foil was used as the receiver at a distance of 12 cm from the syringe needle (Fig. S4, Supplementary Information).

To further get ACP/PLLA composite nanofibrous scaffold, different contents of ACP particles (0%, 5% and 10% on PLLA weight basis) were incorporated in certain concentration of PLLA matrix to form 3 groups of nanofibrous scaffolds, which were named as ACP-free PLLA, ACP-5 wt%/PLLA and ACP-10 wt%/PLLA scaffolds, respectively.

The morphology of electrospinning nanofibrous scaffold was examined with SEM (JEOL JSM-6460LV, Japan). The samples were gold-coated to minimize the charging effect before observation. The micrographs with 8000 × magnification and Image J software (National Institute of Health, USA) were used to evaluate the average diameter of nanofibers.

### *In vitro* hydrolytic degradation

The dried electrospinning samples were cut into square pieces with weight of 0.05 g. They were immersed in centrifuge tubes containing 10 mL of 9% sodium chloride solution. The centrifuge tubes were placed in a constant temperature incubator (37 °C, 160 rpm). At certain time points, the samples were took out and washed with ultrapure water, then dried to constant weight for characterization.

The morphology of nanofibrous scaffolds during hydrolytic degradation was also characterized by SEM. The weight change was calculated as follows: weight change (%) = (W_1_/W_0_) × 100. Here, W_0_ and W_1_ are the weights of samples before and after hydrolytic degradation, respectively. The reported weight change was the average of 3 samples. The molecular weight change of PLLA was tested by GPC (UPLC/Premier, Waters, USA). The samples were dissolved in tetrahydrofuran and polystyrene was used as the standard sample.

The release of calcium ion during scaffold degradation was measured with calcium colorimetric assay kit (Sigma-Aldrich, USA). In this assay, the calcium ion concentration was determined by the chromogenic complex formed by calcium ion and *o*-cresolphthalein, which was proportional to the concentration of calcium ion. A total of 90 μL chromogenic reagent and 60 μL calcium assay buffer were added to 96 well plates containing 50 μL supernatant. The reaction system was incubated in room temperature and protected against light for 5–10 min before absorbance measurement at 575 nm.

The release of orthophosphate ion during scaffold degradation was measured with phosphate colorimetric assay kit (Sigma-Aldrich, USA). Orthophosphate reacted with a chromogenic complex and the formed colorimetric product was proportional to the amount of orthophosphate. A total of 30 μL phosphate reagent was added to 96 well plates containing 200 μL supernatant. The reaction system was incubated in room temperature and protected against light for 30 min before absorbance measurement at 650 nm.

### *In vitro* bioactivity assay

The thawed mouse calvaria-derived preosteoblastic cells MC3T3-E1 (American type culture collection) were incubated in α-MEM supplemented with 10% fetal bovine serum (FBS), 100 U/mL penicillin and 100 μg/mL streptomycin, in a humidified incubator at 37 °C with 5% CO_2_ (Thermo Fisher Scientific, China). The medium was changed every 3 days. Three groups of scaffolds (ACP-free PLLA, ACP-5 wt%/PLLA and ACP-10 wt%/PLLA) were sterilized by exposing in ultraviolet for 4 h, and further used for cell seeding and evaluation.

The cell morphology and distribution on the scaffold were evaluated by SEM. The cell proliferation was tested by cell counting kit-8 (CCK-8, Dojindo, Japan) assay. The MC3T3-E1 cells were seeded in 12-well tissue culture plate (1.0 × 10^4^ cells/well) containing α-MEM medium plus 10% FBS. The CCK-8 assay was carried out on 1, 4, and 7 days after cell seeding. Cell proliferation was measured in 96-well tissue culture plate following the kit instruction. Briefly, 10 μL CCK-8 color reagent was added to each well and then the plate was incubated at 37 °C for 3 h. The absorbance was read at 450 nm in Varioskan Flash (Thermo Fisher Scientific, USA). All experiments were repeated in triplicate and each group had 3 replicates.

For ALP assay, the MC3T3-E1 cells were co-cultured with nanofibrous scaffold for 7 and 14 days, respectively. Afterwards, the medium was removed. The cells were transferred to centrifuge tube and treated with RIPA (Applygen, China) for 10 min at 4 °C. The lysates were clarified by centrifugation. The supernatant was collected for ALP assay by measuring the release of p-nitrophenol at 405 nm using ALP activity kit (Zhongsheng, China). The amount of ALP in the cells was calculated according to the formula provided by the kit. The ALP activity was normalized by total protein content that was quantified by BCA protein concentration assay kit (Beyotime Biotechnology, China).

### *In vivo* bioactivity assay

All experiments involving the use of animals were in compliance with Provisions and General Recommendation of Chinese Experimental Animals Administration Legislation and were approved by Beijing Municipal Science & Technology Commission (Permit Number: SCXK (Beijing) 2006–0008 and SYXK (Beijing) 2006–0025). Adult wild-type Sprague Dawley male rats were purchased from Vital River Corporation (China). The animals were divided into 3 groups randomly according to the implanted nanofibrous scaffolds: (1) ACP-free PLLA group, (2) ACP-5 wt%/PLLA group, (3) HA-5 wt%/PLLA group. A 5 mm craniotomy defect was created on the parietal calvarial bone. After careful haemostasis, the cell-free scaffolds were placed to cover newly generated bone defects and the skin was closed with sutures. Animals were sacrificed and calvarial bones were harvested at 8 weeks for ex vivo end point analysis by Micro-CT and histology (n = 3). For Micro-CT test, the calvarial bone specimens were fixed in 10% formalin for 2 days and incubated in 70% ethanol. The samples were then scanned and analyzed using Micro-CT (Inveon MM system, Siemens, Germany). For histological analysis, the calvarial bone was decalcified with 10% EDTA for 2 weeks, dehydrated through series of ethanol, embedded in paraffin, and sectioned at a thickness of 5 μm. The sections were stained with H&E.

### Statistical analysis

Data were presented as mean ± standard deviation (SD). One-way analysis of variance (ANOVA) was used to test the between-group differences. Statistical significance was determined as *p* < 0.05.

## Additional Information

**How to cite this article**: Niu, X. *et al*. Sustained delivery of calcium and orthophosphate ions from amorphous calcium phosphate and poly(L-lactic acid)-based electrospinning nanofibrous scaffold. *Sci. Rep.* **7**, 45655; doi: 10.1038/srep45655 (2017).

**Publisher's note:** Springer Nature remains neutral with regard to jurisdictional claims in published maps and institutional affiliations.

## Supplementary Material

Supplementary Information
