# Supplementary material for: Sustained delivery of calcium and orthophosphate ions from amorphous calcium phosphate and poly(L-lactic acid)-based electrospinning nanofibrous scaffold
Source: Sci Rep. 2017 Mar 31;7:45655. doi: 10.1038/srep45655 (PMC5374505; doi:10.1038/srep45655)
Supplement: Supplementary Information [file srep45655-s1.pdf]

Supplementary Information

Sustained delivery of calcium and orthophosphate ions from amorphous calcium phosphate and poly(L-lactic acid)-based electrospinning nanofibrous scaffold

Xufeng Niu, Zhongning Liu, Feng Tian, Siqian Chen, Lei Lei, Ting Jiang, Qingling Feng, Yubo Fan

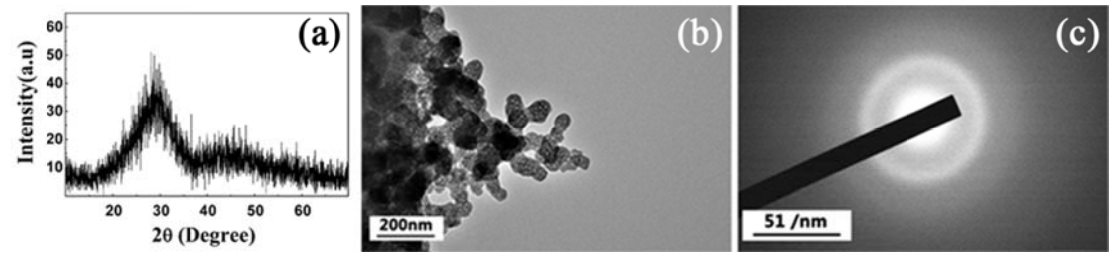

Fig. S1. XRD pattern (a) and TEM micrographs (b and c) of the prepared ACP sample. The broad diffraction of XRD pattern was consistent with amorphous structure of ACP particle. TEM images showed the spherical precursor, which could be defined as ACP, since this unstable status of calcium phosphate had a typical diffraction pattern of amorphous halo ring.

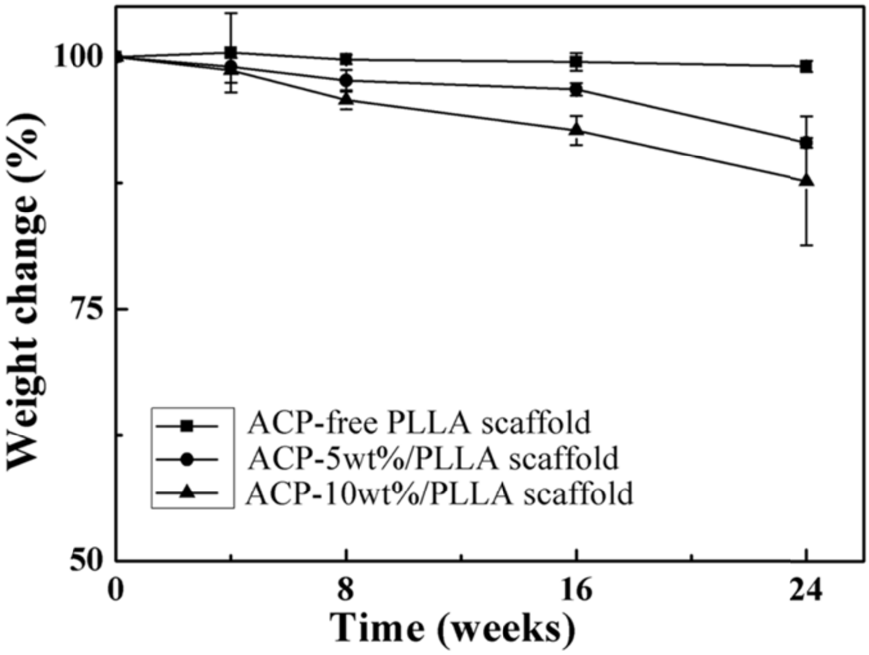

Fig. S2. Weight loss of ACP/PLLA electrospinning nanofibrous scaffold with different contents of ACP particles during 24 weeks of *in vitro* hydrolytic degradation.

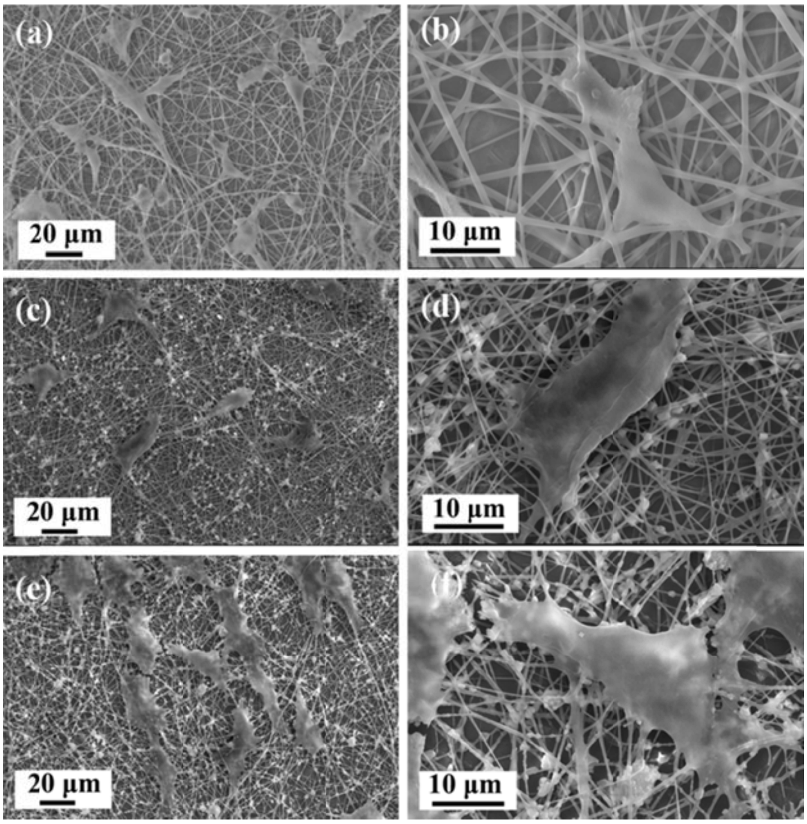

Fig. S3. SEM micrographs of MC3T3-E1 cells cultured with ACP/PLLA scaffold for 7 days. (a)-(b): Cell adhesion and distribution on ACP-free PLLA scaffold. (c)-(d): Cell adhesion and distribution on ACP-5wt%/PLLA scaffold. (e)-(f): Cell adhesion and distribution on ACP-10wt%/PLLA scaffold.

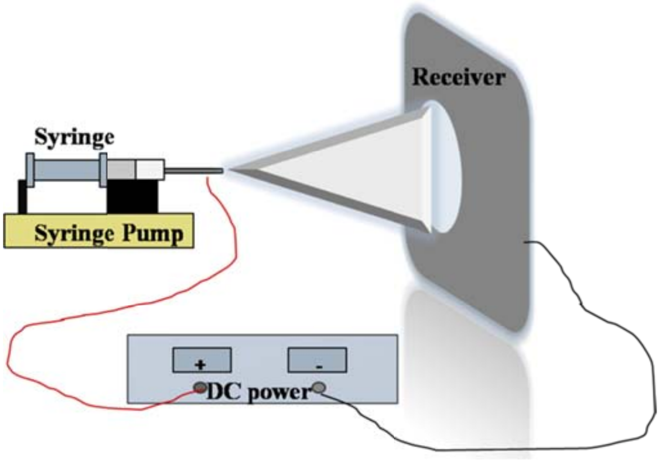

Fig. S4. Schematic representation of PLLA-based electrospinning nanofibrous scaffold. Receiving distance of 12 cm and voltage of 20 kV were loaded between syringe needle and receiver.
